# Material and Topology Optimization of Composite Bone Plate to Reduce the Stress Shielding Effect

**DOI:** 10.3390/ma19102082

**Published:** 2026-05-15

**Authors:** Krzysztof Szymkiewicz

**Affiliations:** Faculty of Mechanical Engineering, Cracow University of Technology, 24 Warszawska, 31-155 Kraków, Poland; krzysztof.szymkiewicz@pk.edu.pl

**Keywords:** bone fixation plate, PEEK-CF polymer composite, finite element method, topology optimization

## Abstract

Bone fractures are often treated using invasive methods involving osteosynthesis plates. These plates are typically made of metallic materials such as titanium or steel. However, their high stiffness relative to bone tissue can contribute to the undesirable stress shielding effect. Therefore, there is a growing interest in developing new, more friendly biocompatible materials with improved mechanical properties. A promising candidate is a polymer composite made of high-strength PEEK reinforced with carbon fibers, which was the subject of this study. The aim of this work was a numerical analysis of osteosynthesis plates made from conventional materials and from PEEK-CF composite. The study also included geometric modification of the composite plate using topology optimization methods to reduce the stress shielding effect. The obtained results confirmed that the use of a geometrically optimized composite osteosynthesis plate can reduce bone unloading and ensure an appropriate stress distribution in the implant–bone system.

## 1. Introduction

The human skeleton, also known as the musculoskeletal system, is the supporting structure of the body and has many important functions, including giving the body its shape, implementing movement, protecting organs, storing minerals and helping to form blood cells. The proper functioning of the system is sometimes disrupted by short-term mechanical overload or osteoporotic changes in the bone tissues that lead to disruption of their continuity. Bone fractures are a common phenomenon, whose treatment and proper tissue healing are time-consuming and require a specialized approach [1,2]. In these cases, the cracked bone is usually stiffened with the help of an osteosynthesis plate with given dimensions and shape [3]. This approach relieves the damaged tissue, which promotes bone healing processes. Bone fracture plates are produced from metallic materials like titanium alloys or stainless steel [4]. However, the high difference in stiffness modulus between metallic alloys and bone tissue can contribute to the occurrence of stress shielding and uneven stress distribution in the bone, as well as the loosening of the implant. It could lead to pain reactions and abnormal bone growth around the fracture.

Therefore, more friendly materials with mechanical properties similar to bone tissue are elaborated. Good candidates could be composites such as glass fiber (GF) or carbon fiber (CF) reinforced polymer [5]. Various materials have been analyzed so far, including GF/PP, CF-PEEK and CF/GF-epoxy composites. These materials are dedicated for biomedical applications mainly because of biocompatible properties.

So far, components used for stabilizing fractured bones have been analyzed using various materials, including both metallic and composite materials [6,7]. The effect of using metallic plates made of titanium and magnesium alloys on the mechanical properties of the bone–plate connection has been studied using numerical methods. These studies have shown that metallic materials have a lower load transfer capability compared to polymer composites [8]. It appears that a plate made of a polymer material such as PEEK reinforced with carbon fibers should provide lower stress levels, thereby reducing the stress shielding effect in comparison to conventional plates.

For the applications under consideration, an important issue besides the structural and mechanical properties of bone plates is the connection at the implant–bone tissue interface. In this case, minimizing the deformations of the plate, as well as limiting the formation of stresses and deformations of the bones that may adversely affect the stiffening of the damaged tissue and its healing processes is expected.

Optimization of composite plates structure allows to obtain the appropriate mechanical properties for dedicated application. The properties of composite structures are depend on the arrangement of the reinforcing phase in the material. From the other side an important stage of composite modeling is topological optimization, which allow to reduce the weight of the model for given boundary conditions. A multivariate approach to the mechanical analysis and optimization of such parts is key to obtaining materials with the requirements demanded, including the minimisation of stresses and strains at the implant–bone tissue interface.

It should be noted that both material and structural analysis are key issues in the study of biomechanical systems [9]. The appropriate selection of implant material and its shape affects the stress and strain distribution within the analyzed system. In such cases, contact interactions between individual components of the system are also taken into account. Attempts to model and describe various biomechanical systems have been undertaken and reported in the literature [10,11]. Nevertheless, this topic, particularly in terms of describing the behavior of biomechanical systems such as those used to stabilize bone tissue, remains an area of ongoing research [12].

Therefore, the aim of the paper is modeling and structure optimization of a bone stabilizing plate built of carbon fiber-reinforced polymer composite. It was supported by topological optimization leading to finding the optimal structure of a low-mass plate. Such an optimized plate can perform both the functions of stiffening the fractured bone and forming acceptable stresses and deformations at the plate–bone interface.

Numerical studies have been performed for load system and boundary conditions close to real cases of bone fractures. Modeling and numerical simulations have been realized with the use of the ANSYS Workbench 2021 R2 software.

## 2. Materials and Methods

### 2.1. Research Approach

The investigations were divided into four steps comprising the modeling of the ostheosynthesis plate and fractured bone–plate connection system, the numerical simulation of the model, the topology optimization of the plate and also the analysis of the stress shielding factor. Numerical simulations were performed for the plate made of polymer composite and metallic materials which are commonly used for such applications.

### 2.2. Creating a Model of the Plate, Bone and Connecting Screws

The bone plate used to stabilize fractures of the tibial bones was designed using the SpaceClaim module in ANSYS Workbench 2021 R2. The plate is a segment of the lateral surface of a cylinder with a diameter of 29 mm. Its length and thickness were 103 mm and 4 mm, respectively, while the radius of curvature was 13.7 mm.

The bone model, consisting of an outer cortical part and an inner cancellous part, was simplified into the form of a cylinder with a diameter of 25 mm. The proposed approach to geometric modeling of the bone was adopted to preserve the global stiffness of the model and to isolate the effect of the plate on stress redistribution. This solution enabled a detailed assessment of changes in mechanical strength parameters as well as the stress shielding effect.

The applied modeling simplification was adopted to ensure controlled and repeatable conditions of analysis for all considered material and geometric variants of the plate. This approach makes it possible to reduce the influence of anatomical variability and to assess the effect of the plate’s properties and structure on stress distribution and load transfer within the system, which was the aim of this study.

The fracture was defined as a transverse discontinuity in a plane perpendicular to the longitudinal axis of the bone. A solid body was introduced between the two bone segments within the fracture gap to represent the callus tissue forming during the healing process. The considered fracture in the bone model was stabilized using a plate and screw construct following an open reduction and internal fixation (ORIF) approach. The osteosynthesis plate was positioned along the surface of the bone and secured with screws inserted above and below the fracture site, in the proximal and distal parts of the bone model.

The screws connecting the fractured bone to the osteosynthesis plate were designed without modeling threads. Their length was 26 mm, and their diameter was 4.5 mm. The screws pass through the entire thickness of the bone model, stabilizing it on both sides. The prepared models were assembled to obtain the final form of the fractured bone model stabilized with an osteosynthesis plate fixed with screws.

### 2.3. Material Properties

The study analyzed two types of bone plates with a composite structure, made from the polymer material polyetheretherketone (PEEK) reinforced with carbon fiber at volume fractions of 10% and 30%, which were referred to in the study as PEEK-10CF and PEEK-30CF, respectively.

Additionally, for comparison purposes, plates made from three conventional materials commonly used for such components were also studied, including titanium alloy, magnesium alloy, and stainless steel. Regardless of this, a functionally graded metal–ceramic FGM Ti-HAP plate was also considered. The FGM plate was characterized by a linear variation in chemical composition, and its properties were determined based on the Power Law. The screws were composed of stainless steel. In the study, the tibial bone was considered, consisting of an outer cortical part and an inner trabecular part. The cortical bone is characterized by high strength and stiffness and is primarily responsible for load bearing. Additionally, it exhibits anisotropic properties, meaning its mechanical behavior depends on the direction of analysis. The trabecular part of the bone has a spongy structure, and its mechanical properties, such as strength and hardness, are lower than those of the cortical bone. It also has a density approximately five times lower than the outer part of the bone. The trabecular bone, titanium, magnesium alloy, stainless steel, PEEK-10CF, and callus are assumed to exhibit isotropic elastic behavior, while PEEK-30CF and the cortical bone are modeled as anisotropic materials.

The analysis was performed assuming a linear-elastic constitutive description for all considered materials. For cancellous bone, titanium alloy, magnesium alloy, stainless steel, PEEK-10CF composite, and callus tissue, which are isotropic materials, the stiffness matrix was formulated using the material constants of Young’s modulus and Poisson’s ratio. Thus, homogeneous, direction-independent material properties were assumed. The cortical bone model and the PEEK-30CF composite plate were analyzed as an orthotropic model, using nine elastic constants: Ex, Ey, Ez, Gxy, Gyz, Gxz, vxy, vyz, vxz. For the PEEK-30CF polymer composite, the coordinate system was defined such that the first direction aligns with the predominant orientation of the carbon fibers, while directions 2 and 3 are perpendicular to it. In the case of the cortical bone, a local material coordinate system was considered in which the first direction coincides with the principal axis of the bone, i.e., the longitudinal direction. This direction corresponds to the dominant load-bearing axis. The remaining coordinate directions are orthogonal and defined as transverse directions. The choice of the adopted material axis system reflects the anisotropic nature of cortical bone. Taking this into account, the stress–strain relationship is defined using the general form of Hooke’s law for an orthotropic material.

The properties of the callus tissue corresponded to a state of 50% healing of the fractured bone. The mechanical and physical properties of the used materials of the bone and osteosynthesis plate are presented in Table 1.

### 2.4. Boundary Conditions of Mechanical Analysis of Fractured Bone–Plate Connection System

Based on the preliminary numerical analysis, it was determined that the models of the bone, callus, plate, and screws should be built with element sizes of 2 mm, 0.2 mm, 0.5 mm, and 1 mm, respectively, which allows for achieving optimal mechanical results with the appropriate convergence. Therefore, these element sizes were implemented in the mesh model during all subsequent studies. The analyses were conducted for the case without a contact connection between the plate and the bone. The distance between the lateral surface of the bone and the plate was 1 mm. In the considered model, loads were primarily transferred through the osteosynthesis plate and the system of screws fixing it. The adopted contact assumption at the plate–bone interface corresponded to a configuration typical of locked plating osteosynthesis, in which no compressive contact occurs between the plate and the bone surface. In such a case, load transfer takes place mainly through the screw–plate connection, without direct surface contact between the plate and the bone. This condition was adopted based on previous studies of plates for osteosynthesis made from metal–ceramic functionally graded material (FGM) [7]. To replicate the actual conditions of the considered mechanical system, one end of the bone model was fixed in all directions at the location of the transverse surface. A load was applied to the other end of the bone, with the vector directed along the main axis of the bone model. The analyses were performed for the case of 50% healing of the tibia, and therefore the applied force was 800 N, corresponding to the typical body weight of an adult of approximately 80 kg.

### 2.5. Topology Optimization of Polymer Composite Bone Plate

Finding a material structure with the lowest possible mass that meets the assumed model properties and boundary conditions required the application of a topology optimization process. The method involves an algorithmic optimization of elements, usually through material reduction performed iteratively. The optimization processes were carried out based on the Solid Isotropic Material with Penalization (SIMP) method. Topology optimization was performed assuming three different volume retention variants of the model body: 75%, 50%, and 25%. In the subsequent part of the study, the optimized models were designated as TOPO75, TOPO50, and TOPO25. The optimized models were subjected to numerical analysis under the same boundary conditions as the initial models.

Furthermore, the geometry of the model exhibiting the best mechanical response in the plate–bone tissue system was additionally modified to meet all manufacturing requirements, taking into account production by 3D printing. To verify the applied geometric correction, a new numerical simulation was also performed for the system using this revised model.

### 2.6. Stress Shielding Factor

The SSF coefficient was determined to assess the loading of the considered mechanical system with respect to the potential formation and development of stress shielding in real cases. Based on this, attempts were made to minimize it using topology optimization methods. The coefficient was calculated from the equivalent stresses determined according to the von Mises–Hencky hypothesis for the bone model with and without stiffening. This allowed the coefficient to be calculated using the following formula:

$$SSF=1-\frac{\sigma_{fixed\;bone}}{\sigma_{non-fixed\;bone}\;}$$

It is generally assumed that if the coefficient exceeds a value of 0.5–0.6, excessive unloading of the bone may occur, which can consequently lead to stress shielding and bone resorption. Lower values indicate that the bone carries part of the load, and such a range of the SSF coefficient is considered acceptable. However, this threshold should be assumed as approximate rather than strictly defined.

The SSF parameter indicates the level of stress reduction in bone tissue when stabilization is achieved using a plate. In other words, it defines the proportion of load carried by the bone in relation to the implant. It should be noted that the SSF coefficient does not have strictly defined threshold values. Therefore, the indicated range should be considered approximate. For example, an SSF value of 0.5 indicates a 50% reduction in stress within the bone tissue, meaning that half of the load is carried by the implant. In this case, an SSF range of 0.5 to 0.6 may indicate a significant unloading of the bone and a substantial contribution of the implant to load bearing. From a biomechanical perspective, increased unloading of the bone is not desirable due to the reduction in mechanical stimuli, which may hinder the healing process and consequently lead to bone resorption. It is expected that in the initial state of treatment the implant should carry a greater portion of the load, and then, as the bone tissue heals, the amount of load transferred to the bone should gradually increase.

The variability of the SSF coefficient depending on the degree of bone healing was also analyzed. The boundary conditions, including loading and the properties of the callus tissue, were determined based on biomechanical basics [2,13]. This analysis allowed for the assessment of changes in load transfer over time, as well as the stabilization of the system. The adopted boundary conditions, including loading and the properties of the callus tissue, are presented in Table 2.

## 3. Results

### 3.1. Effect of Mesh Size on the Mechanical Response of Plate–Bone System

**Figure 1:**
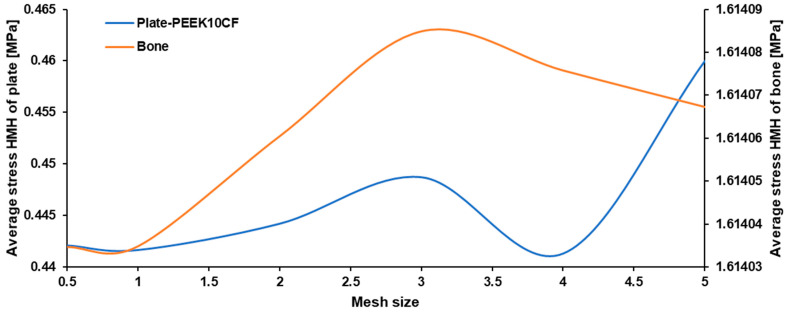
Effect of mesh density on the maximum Huber Mises Hencky HMH stress in the plate–bone system.

To achieve high-accuracy results, a convergence analysis was performed by evaluating the influence of mesh element size for both the plate and bone on the mechanical response of the system. A series of numerical simulations was carried out using different mesh densities obtained through mesh refinement. For each case, the average stress value of the two analyzed models was determined. The obtained results were plotted as a function of average stress versus element size (Figure 1). The conducted analyses showed that increasing the element size led to significant discrepancies in the obtained results, whereas smaller element sizes tended to converge to a stable range of values. The smallest analyzed element sizes (0.5 and 1) allowed for the achievement of result convergence, indicating that the mesh was sufficiently accurate. The obtained results were subsequently used in the remaining numerical simulations described in this study.

### 3.2. Effect of Plate Material on Mechanical Properties of Plate–Bone System

Numerical analysis of a stiffened bone model using a plate made of conventional materials and PEEK-CF composite showed that the model undergoes the largest deformations and von Mises stress in the case of conventional solutions with metallic plates. The maximum stress values were approximately 33 MPa for the model with a steel plate, while the use of a titanium or magnesium alloy plate resulted in a decrease in stress to around 24 MPa. The most favorable stress distribution occurs in the model with a plate made of polymer composite, reaching values of approximately 22 MPa. In all analyzed cases, the deformations of the model were small, ranging from 0.04 mm to 0.07 mm, which is acceptable for such solutions. The average strain of the system, determined for both conventional and composite plates, was also small, around 9 × 10^−5^ mm/mm. A summary of the maximum deformation, stress, and average strain results for the plate–bone system is presented in Table 3.

**Figure 2:**
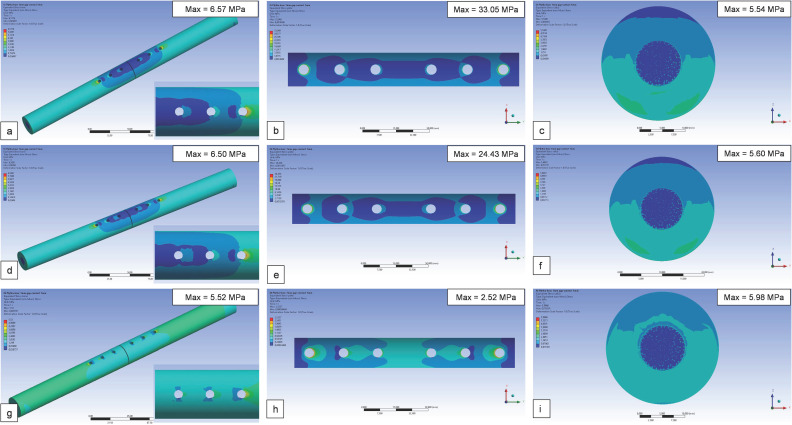
Stress distribution on a model of a bone, a plate and a callus for the system with plate made of: (**a**–**c**) steel, (**d**–**f**) titanium alloy, and (**g**–**i**) PEEK-10CF composite.

The stress distribution maps according to the Huber–Mises–Hencky criterion are shown in Figure 2, observed in relation to the components of the model, including the bone, the plate, and the callus tissue model, and considering the plates made of steel, titanium alloy, and 10CF-PEEK polymer composite.

On the surface of the bone model, it can be observed that in its central part, near the fracture, the lowest stress values are formed, which then increase in both the distal and proximal directions of the bone. The highest stresses were observed at the extreme locations of the holes designed for attaching the plate to the bone with screws. Generally, in the area covered by the plate, only small stresses can be noted. Nevertheless, in the case of models with metallic plates, the stress distribution in the bone is not uniform, in contrast to the model with a composite plate, where the stress values formed are low.

The maximum stresses on the bone with a steel or titanium plate were approximately 6.5 MPa, while for the bone with a composite plate, they were slightly lower. The composite plate model showed the most favorable stress distribution, and the recorded stress values were approximately 10 and 13 times lower compared to the titanium alloy and steel plates, respectively. The observed maximum stress values in the callus tissue were similar in all cases, around 6 MPa. Lower stresses accumulated on the side of the bone–plate connection, while higher stresses were observed on the opposite side.

**Figure 3:**
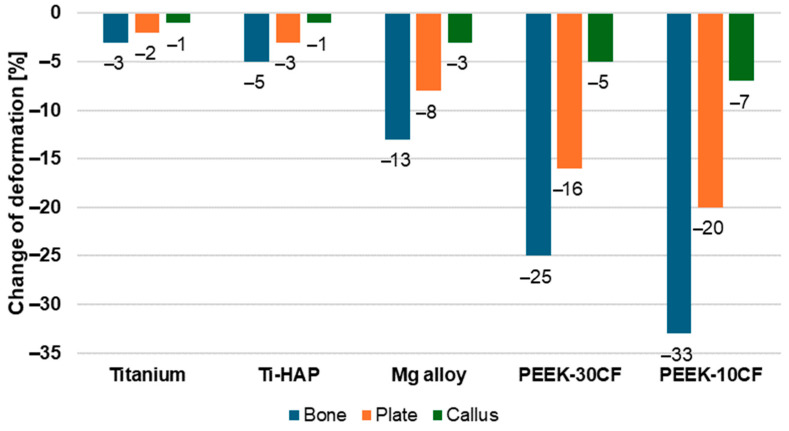
Deformation changes of bone–plate model compared to steel plate system.

The percentage changes in deformation values observed in the bone, plate, and callus tissue, relative to the model with a steel plate, are shown in Figure 3. In all cases considered, a decrease in deformation is observed, which varies depending on the material from which the plate is made. The use of titanium and Ti-HAP composite plates for osteosynthesis leads to a slight reduction in deformation of approximately 2–3%. An implant made of a lightweight magnesium alloy results in a greater reduction in stress in the bone and plate by 13% and 8%, respectively. The largest changes in deformation were observed for models with a PEEK plate reinforced with carbon fiber. It was observed that the callus tissue exhibits the smallest decreases in deformation, ranging from 1% to 7% compared to the model with a steel plate.

**Figure 4:**
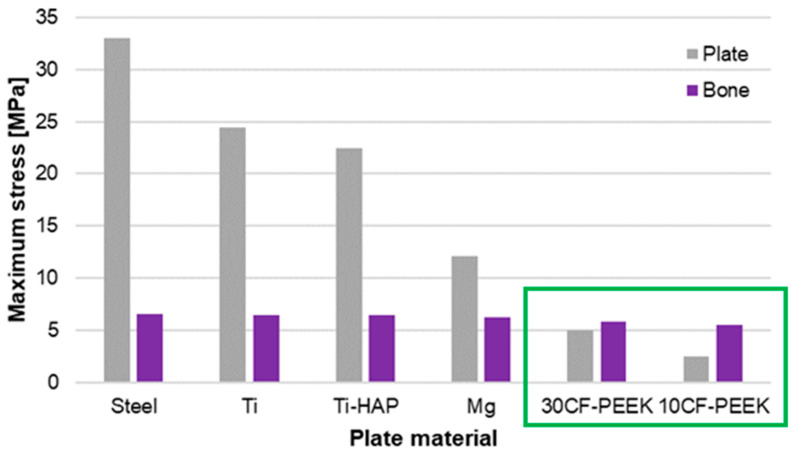
Maximum HMH equivalent stress for the plate and bone model.

The analysis of maximum stresses for the osteosynthesis plate and bone showed that the largest differences in this parameter were obtained for the model with steel and titanium plates, with values as follows: 33 MPa and 24 MPa for the plate, and approximately 6.5 MPa for the bone (Figure 4). Lower stress values and smaller differences between the plate and bone were observed for the model with a magnesium alloy implant. In the case of the system with a composite plate, higher stress values were obtained for the bone relative to the plate, which could be beneficial from the perspective of the biomechanical system and the bone tissue healing processes.

### 3.3. Topology Optimizaton Effect of Composite Plate on the Geometry and Mechanical Properties of the System

**Figure 5:**
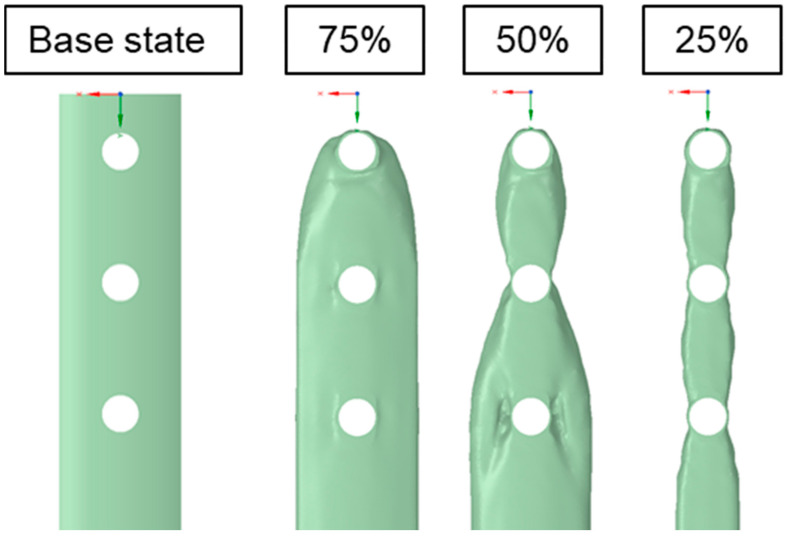
Geometry of the osteosynthesis plate in the state before and after topology optimization.

Topology optimization of the osteosynthesis plate model enabled the removal of material volume from regions subjected to low mechanical loads and stress concentrations, thereby reducing the final weight of the component (Figure 5).

It was observed that the first stage of optimization, with 75% of the material volume retained, primarily resulted in the rounding of the plate ends, with material being removed in these areas. The subsequent optimization iteration showed that material could be removed in the regions of the outermost screw holes without affecting the structural performance of the system. The final stage of the iteration, in which up to 75% of the material volume was considered for removal, led to a significant reduction in the plate width compared to the initial state, as well as a slight widening of the plate in the regions between individual screw holes.

Table 4 presents the obtained values of maximum deformation and stress for the bone model and the composite plate at each stage of the topology optimization process. In all cases, similar deformation values were obtained for the bone model and the plate, amounting to approximately 0.04 mm and 0.02 mm, respectively. The maximum stresses in the bone exceeded 5 MPa, while the lowest value was obtained for the TOPO25 plate after optimization.

**Figure 6:**
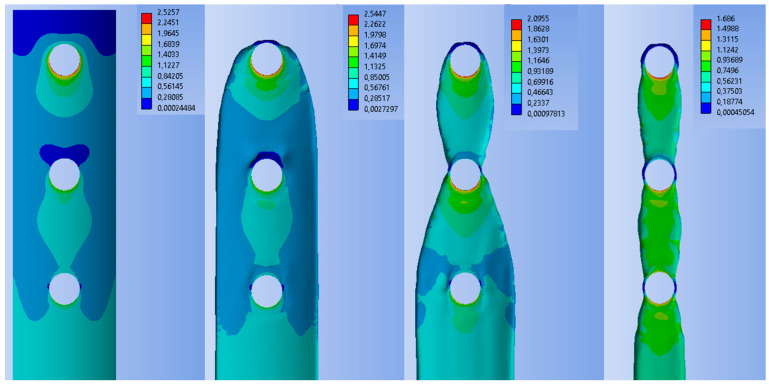
Distribution of HMH equivalent stresses in the PEEK-CF plate in the initial and post-topology optimization states.

Figure 6 shows the stress distribution on the PEEK-10CF composite plate before and after topology optimization, presented from the bone–implant interface side. It was observed that, in all cases, the highest stresses occur at the screw holes and decrease toward the central region of the plate. The TOPO25 plate exhibited the lowest stress values. It should be emphasized that, for this model, the stress distribution was the most uniform, in contrast to the other models where more abrupt stress changes were observed.

**Figure 7:**
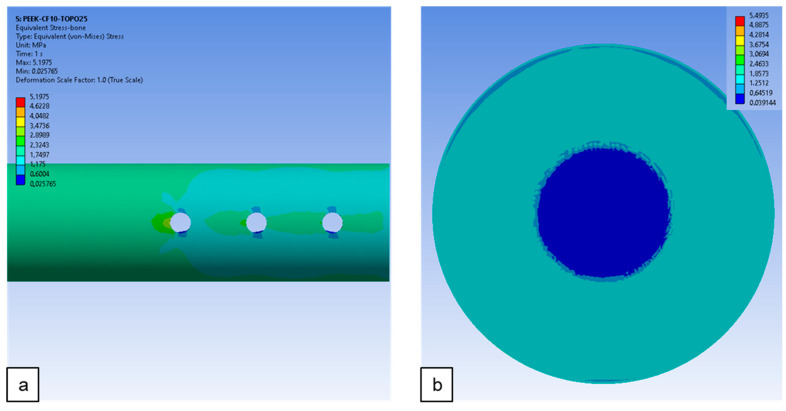
Equivalent HMH stress distribution maps of the bone (**a**) and callus tissue (**b**).

Figure 7 presents the stress distribution maps for the bone model and the callus tissue. The stress distribution and the maximum values obtained for the model were similar to those observed in the case of the initial PEEK-CF composite plate. In the callus tissue, within the cancellous bone region, relatively low stresses were recorded, which then slightly increased toward the outer part of the bone model.

**Figure 8:**
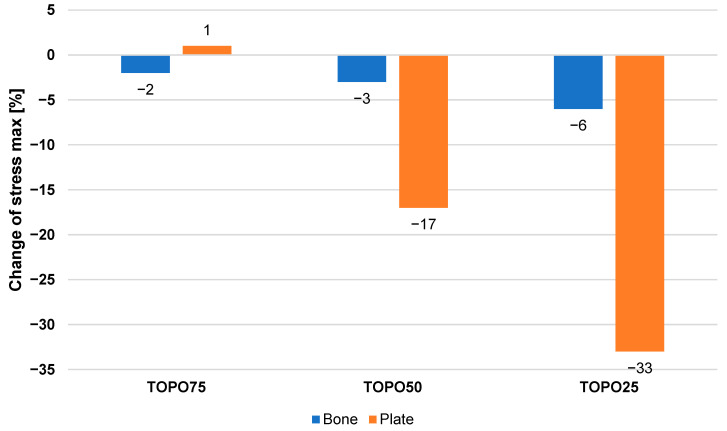
Percentage change in the maximum stress in the bone and the plate after topology optimization relative to the system with the initial composite plate.

Figure 8 shows the percentage changes in the equivalent maximum stress generated in the bone and the plate after each stage of topology optimization, relative to the initial configuration of the CF-PEEK composite plate. Topology optimization with a material volume fraction of 25% resulted in the largest changes in the stresses within the model. These changes amounted to 6% and 33% for the bone and the osteosynthesis plate, respectively.

For the remaining cases, the changes were smaller, while for the model with the plate showing the smallest volume reduction (TOPO75), the stress changes were within the measurement error range. A slight difference in the analyzed parameter between the plate and the bone was observed for the initial model, which could indicate a more favorable structural solution in relation to the biomechanical system.

Nevertheless, in the case of the TOPO25 plate, it is possible to significantly reduce the amount of material used, which constitutes a major advantage compared to the other material scenarios.

### 3.4. Evaluation of the Influence of Technological Constraints on the Mechanical Response of the Plate–Bone System After Geometry Modification

**Figure 9:**
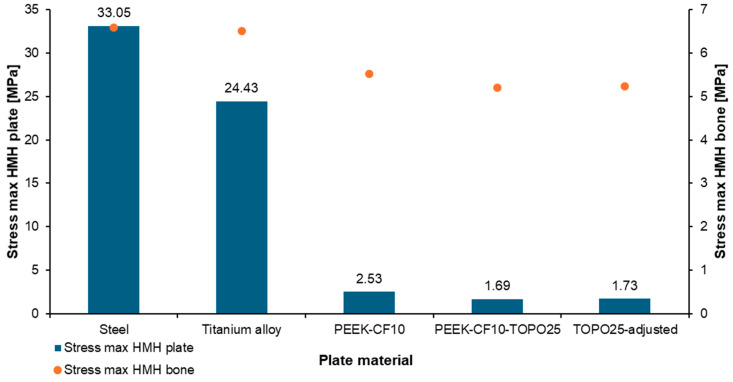
Effect of plate material on the maximum HMH stress of model.

As indicated by the topology optimization process and numerical analysis, the composite PEEKCF10-TOPO25 plate appears to be the most suitable geometric solution for the considered biomechanical system. However, in order to verify the potential manufacturability of such a plate, the geometry was modified to account for technological constraints, including wall thickness and potentially critical regions. A repeated numerical analysis of the plate–bone system showed that the considered loading scheme leads to the development of similar maximum stress values in both the plate and the bone model (Figure 9). Compared to the system with the composite plate prior to topology optimization, the recorded stresses were lower by just under 1 MPa. These values are more favorable in comparison to systems using plates made of conventional materials.

**Figure 10:**
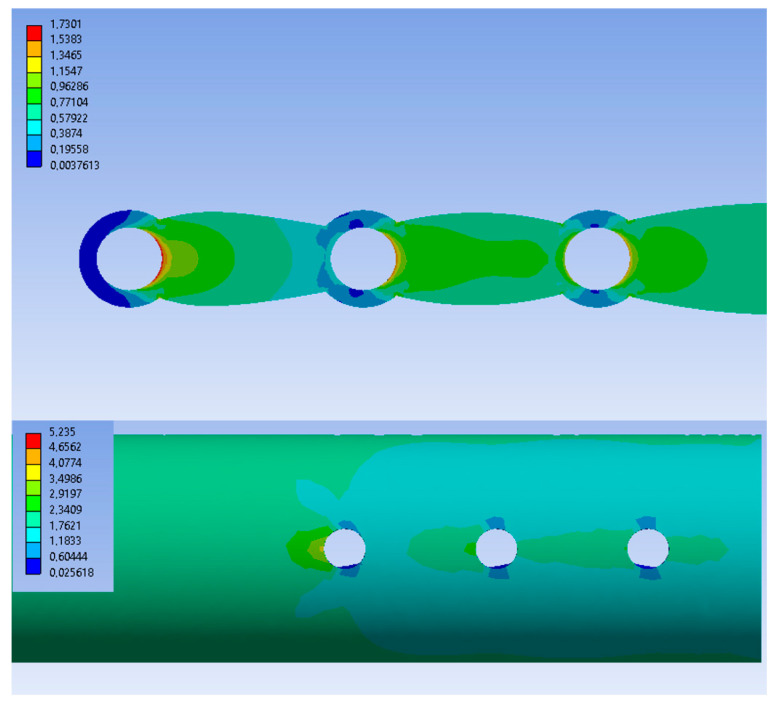
HMH stress distribution maps on the bone and PEEK-CF10-TOPO25-adjusted plate.

Figure 10 presents the maps of von Mises (HMH) equivalent stress distribution formed in the TOPO25-adjusted plate after geometric correction, as well as in the bone model. The observed stress distribution is similar to that of the model with the plate prior to the aforementioned correction (i.e., with the PEEK-CF10-TOPO25 composite plate).

### 3.5. Analysis of the Stress Shielding Factor

**Figure 11:**
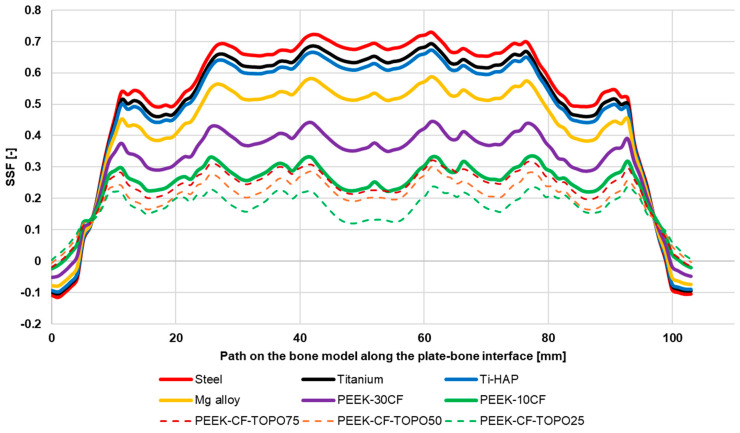
Graph of SSF coefficient variations along the fractured bone at the site stabilized with a plate.

Changes in the SSF coefficient along the fractured bone in the area stabilized with a fixation plate are illustrated in Figure 11. It can be noted that the highest SSF values were obtained for conventional plate material solutions. In all cases, a similar variation in the coefficient along the analyzed path on the bone was observed. Local decreases in the SSF coefficient were noted, occurring at the locations of the screws connecting the bone to the plate. The use of a stiffening plate made of composite material allowed for a significant reduction in the SSF coefficient, reaching values acceptable with respect to the load applied to the analyzed biomechanical system. For the PEEK-10CF plate, the obtained SSF coefficients ranged on average from 0.2 to 0.3. Topological optimization of the composite plate design led to a further reduction in the SSF coefficient, with the greatest reduction observed for the variant with the highest material reduction—TOPO25.

**Figure 12:**
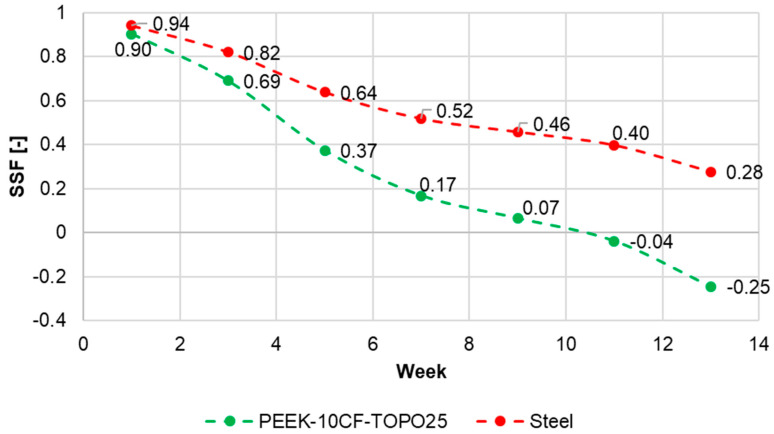
Graph of the SSF coefficient distribution for the steel plate model and the PEEK-CF-TOPO25 model at different time points after fixation of the fractured bone.

Figure 12 shows a graph of the stress shielding factor (SSF) variation over time from the moment of bone fixation. The analysis of the system with the TOPO25 composite plate and the steel plate indicated that during the initial weeks after surgery, similar SSF values were obtained. Subsequently, these values decreased, with the favorable SSF coefficient already achieved for the composite plate model between the 3rd and 4th week, while for the steel plate it occurred around the 6th week. The decreasing trend of the SSF coefficient progressed faster in favor of the composite plate. By the 9th week after bone fixation, the TOPO25 model exhibited an SSF value of approximately 0, while the steel plate model maintained a value of nearly 0.5. In the later stage of the analysis, negative values of the SSF coefficient were also obtained. This would indicate a condition in which the stresses generated in the bone are higher than the reference level, suggesting local overloading. However, it should be emphasized that in the later phase of bone healing, this can be regarded as a beneficial phenomenon, defined as the gradual transfer of load to the bone.

## 4. Discussion

Bone fractures require appropriate intervention to support the healing processes of the damaged bone tissues. Sometimes, surgical interventions are necessary to stabilize the bones, usually by using osteosynthesis plates. These components allow for proper stabilization of the created tissue discontinuity, relieve stress, and ensure the correct processes of bone structure growth. The metallic alloys commonly used for manufacturing these plates can negatively affect the biomechanics of the implant–bone system and the healing processes due to their high stiffness compared to bone. This can result in the phenomenon of stress shielding, leading to tissue atrophy.

Numerous studies focused on analyzing other potential material variants have shown that composite materials appear to be promising. A good candidate could be the polyetheretherketone (PEEK) composite reinforced with fibrous material, which has mechanical properties relatively similar to bone and also exhibits very good biocompatibility. The use of polymer composite materials in biomedical engineering has also been considered for orthopedic solutions (plates, screws, orthoses) as well as in dentistry (implants, prosthetics, splints, dental braces). However, existing research achievements indicate a continuous need for further analysis of composite materials for medicine aimed at optimizing the structural and material properties of implants, contributing to the proper occurrence of bone tissue regeneration processes and the functioning of the implant–bone system. Therefore, this study attempted a staged numerical material analysis, focusing on an orthopedic plate for bone fractures. The research involved topological optimization and examined changes in the stress shielding factor (SSF) depending on the material solution of the plate and the bone healing time. The numerical studies conducted within this work were performed for the case of pure axial compression with a force corresponding to the body mass of an adult patient equal to 80 kg, i.e., 800 N. The adopted model is simplified, as it does not consider other complex loading scenarios. Nevertheless, the primary aim of the study was to analyze the influence of plate material and to optimize its geometry with respect to the mechanical response of the plate–bone system, while the adopted boundary conditions enable a valid comparative evaluation for the considered loading case.

Furthermore, a simplified cylindrical model of the tibia was used, which only partially reflects the actual anatomy of bone tissue. This approach was additionally justified by the location of the considered fracture in the tibial shaft, whose shape can be approximated as cylindrical geometry. The adopted simplification may influence local loading conditions and lead to a slight smoothing of stress concentrations characteristic of complex anatomical geometries. Therefore, the obtained results should be interpreted primarily in a comparative sense, as an analysis of relative differences between the considered implant plate models. It should be emphasized that the simplified bone geometry used is widely applied in comparative biomechanical analyses of orthopedic implants, including optimization studies. This allows for the verification of the conducted analyses and obtained results. The literature shows that the use of such geometric simplifications enables a reliable assessment of changes in stress distribution, load transfer, and trends related to the phenomenon of stress shielding, while limiting the influence of anatomical variability [14,15,16].

In the conducted numerical studies, a case with a gap between the plate and the bone was analyzed. Such a configuration may influence the load transfer mechanism within the considered system. For the adopted setup, the dominant contribution to load transfer is carried by the plate and the screw, which may lead to a partial stress shielding effect due to reduced loading of the bone tissue. When comparing this configuration with a direct implant–tissue contact variant, an increase in stresses within the plate and a reduction in local stresses in the cortical bone can be expected. In the past, different types of plate–bone connections have been analyzed, including configurations with and without contact [7,17,18,19]. As suggested by previous studies, the contact conditions at the plate–bone interface influence the stress distribution in the stabilized bone tissue. Both types of connections lead to different biomechanical conditions in fracture fixation of bone structures. Limiting the surface contact between the plate and the bone may be more favorable from the perspective of load transfer and bone remodeling processes.

### 4.1. Effect of Plate Material on the Implant–Bone Tissue Biomechanical System

Numerical simulations have shown that lower reduced stresses form in the composite plate compared to plates made of metallic alloys. Additionally, the differences in stresses between the osteosynthesis plate and bone are smaller in the case of the composite plate model made of PEEK-CF (approximately 3 MPa) compared to stainless steel and titanium plates, which are 26.5 MPa and 17 MPa, respectively. The obtained results stem from significant differences in the mechanical properties of the analyzed materials, primarily the longitudinal elastic modulus. Metallic alloys have high stiffness (steel ~200 GPa, titanium alloys ~110 GPa), while the longitudinal elastic modulus for the considered CF-PEEK composite reaches approximately 8–20 GPa. This contributes to a more even distribution of loads on the implant and bone.

The obtained results in terms of qualitative or quantitative analysis showed a similar trend to the data in the literature [20,21]. For instance, the analyses conducted by Ceddia et al. indicated slight differences in the determined mechanical parameters compared to our work. However, this could be due to the adopted biomechanical setup and the differences in boundary conditions, including the degree of material properties dependent on the healing stage of the bone [8].

The analyses confirmed that composite plates have an advantage over conventional materials in better adaptation by reducing the stiffness of the plate, which minimizes negative phenomena in the implant–bone system. The use of short-fiber-reinforced composite can improve the stability of the connection. The obtained findings are associated with a more favorable biomechanical environment, which can be appropriate from the point of view of structural efficiency.

### 4.2. Topology Optimization Effect on Mechanical Response of Plate–Bone Tissue System

The optimization of the topology of the composite material PEEK-CF, exhibiting the best mechanical response of the implant–bone tissue system, allowed for the modification of the shape of the plate used in osteosynthesis. It was possible to remove material from areas with a lower contribution to the load transfer while maintaining the appropriate stiffness of the considered model. The optimization process showed that a 75% reduction in the volume of the plate (TOPO25) compared to the initial state ensured acceptable performance results for the model in the analyzed system. The optimized design achieved a material distribution that provided an appropriate stress distribution in critical areas while simultaneously minimizing stress concentrations. A decrease in stresses was noted, specifically by 6% and 33% for the bone and the TOPO25 plate, respectively, compared to the model with the original geometry of the composite plate. The reduction in material had a positive impact both from a biomechanical perspective and from an economic point of view.

Research focused on the structural optimization of plate models for bone fractures has also been described in the literature. It is evident that there is a significant interest in using topological optimization in the design processes of orthopedic implants, considering its impact on the processes of bone growth. A study by Mehboob A. et al. focused on the optimization of plates for mandible fractures revealed that porous and optimized structures can provide better conditions for bone healing processes and improve the biomechanical characteristics of the implant–bone system [22]. Similar analyses aimed at the study of osteosynthesis plates confirmed the positive influence of topological optimization on the distribution of formed stresses in the bone [23]. It was also found that excessive stiffness of plates with typical geometry leads to the undesirable phenomenon of stress shielding, whereas optimization processes allow for the reduction in deformations and distortions in the model [24]. Recent studies have revealed that both the appropriate selection of material and the geometry of the implant influence the function and stability of the plate–bone connection [25].

It should be emphasized that the main advantage of the analyzed composite material, PEEK reinforced with short carbon fibers, is its ability to be processed using additive manufacturing methods (3D printing). This allows for the formation of optimized geometries of the plate. 3D manufacturing techniques have the advantage over others in that they enable control over the formed structure and porosity, which directly influences the stiffness of the material in designated areas. This way, the mechanical properties of the material can be appropriately adjusted for the designed loads and specific anatomical conditions. Additionally, the polymer material PEEK possesses very good biocompatibility and chemical stability, making it a friendly material for implant engineering.

### 4.3. Effect of Bone Stresses on the Phenomenon of Stress Shielding

The analysis of stress formed on fractured and healthy bone allowed for the estimation of the stress shielding factor (SSF). Calculations indicated that the stabilization of fractured bone tissue using a steel or titanium plate for osteosynthesis contributes to a high value of the SSF, which may result in bone resorption, loosening of the implant, or the need for reoperation. The considered gradient metal–ceramic Ti-HAP led to a reduction in this parameter, and better results were obtained for the model with a plate made of a polymer composite. This was confirmed by additional analyses of the variability of the SSF depending on the degree of healing of the bone tissue. The analysis also revealed that optimization of the shape geometry allowed for a reduction in the SSF by approximately 30% for the TOPO25 plate compared to the original PEEK-CF composite plate.

Similar analyses, limited to a qualitative assessment of this phenomenon, have been previously conducted for plates made from conventional materials and composites. It was found that the reduction in the stress shielding phenomenon is dependent on the gap between the bone and the plate [17]. Regarding the material solution for the plates, it was noted that those made from polymer composite can reduce the SS effect [20]. The achievements and results of research described in the literature are consistent with those presented in this work.

However, it should be highlighted that the previously published works mostly did not consider a detailed quantitative analysis of the SSF in the form presented in this paper.

## 5. Conclusions

Based on the numerical research and topological optimization conducted performed for a loading model in the form of pure axial compression, the following conclusions were formulated:

1. The composite plate for bone fractures provides optimal stiffness of the bone–implant system and a more physiological distribution of loads, contributing to improved functional recovery of the bone.
2. An increase in the amount of reinforcing phase results in the formation of higher stresses and deformations in the model, negatively impacting the biomechanical system of the implant and bone.
3. Reducing the volume of the initial PEEK-CF10 by 75% (TOPO25) as a result of topological optimization leads to more favorable biomechanical conditions for the stiffened bone, as reflected in stress and strain distributions.
4. The use of osteosynthesis plates (e.g., polymer or gradient plates) with lower stiffness contributes to the reduction in the stress shielding phenomenon throughout the entire tissue healing period.

In summary, the conducted research indicates the need for further work focusing on additional material and structural analyses. The research carried out and the results obtained in this study are essential for advancing further investigations in this area. A good solution would be to expand the analyses for models with a porous structure made of biocompatible composite materials, which could also be beneficial from a biomechanical point of view. The proposed approach for further research, considering the achievements to date, could influence the development of the discussed topic.

## Figures and Tables

**Table 1:** Properties of materials used for models of bone, osteosynthesis plate, and screws.

No.	Material	Density δ [g/cm^3^]	Young’s Modulus E [GPa]	Poisson’s Ratio ν
1	PEEK-10CF	1.39	8.1	0.35
2	PEEK-30CF	1.38	E_x_ = 21.4 E_y_ = 18.1 E_z_ = 4	V_xy_ = 0.35 V_yz_ = 0.15 V_xz_ = 0.15
3	Ti alloy	4.5	116	0.34
4	HAP	3.15	73	0.28
5	Mg alloy	1.8	45	0.35
6	Stainless steel	7.9	193	0.31
7	Cortical bone	1.8	E_x_ = 7 E_y_ = 18.4 E_z_ = 8.5	V_xy_ = 0.099 V_yz_ = 0.065 V_xz_ = 0.141
8	Trabecular bone	0.37	1.02	0.225
9	Callus (50% bone healing time)	1.43	10	0.3

**Table 2:** Properties of the callus tissue and model loading as a function of healing time.

		Callus		
Week	Force [N]	Density [g/cm^3^]	Young’s Modulus [GPa]	Poisson’s Ratio
1	100	0.5	0.05	0.45
3	300	0.9	0.5	0.42
5	600	1.2	3	0.36
7	800	1.434	10	0.3
9	900	1.6	13	0.28
11	1000	1.75	16	0.27
13	1200	1.9	20	0.25

**Table 3:** Overview of the maximum deformation, stress, and average strain results for the plate–bone system.

Plate Material	Total Deformation Max [mm]	Stress Max [MPa]	Strain Average [mm/mm]
**Steel**	0.0703	33.05	8.47 × 10^−5^
**Ti**	0.0679	24.87	8.64 × 10^−5^
**Ti-HAP**	0.0665	24.75	7.55 × 10^−5^
**Mg**	0.0612	24.08	9.12 × 10^−5^
**30CF-PEEK**	0.0528	22.91	9.76 × 10^−5^
**10CF-PEEK**	0.0474	22.01	1.03 × 10^−4^

**Table 4:** Overview of the maximum deformation and stress results for the bone model and the composite plate before and after topology optimization.

Bone		
Plate Material	Total Deformation Max mm	Stress Max [MPa]
**10CF-PEEK**	0.0474	5.52
**TOPO75**	0.0467	5.42
**TOPO50**	0.0453	5.33
**TOPO25**	0.0435	5.20
		
**Plate**		
**Plate material**	**Total Deformation Max mm**	**Stress Max [MPa]**
**10CF-PEEK**	0.0200	2.53
**TOPO75**	0.0193	2.54
**TOPO50**	0.0192	2.10
**TOPO25**	0.0192	1.69

## Data Availability

The original contributions presented in this study are included in the article. Further inquiries can be directed to the corresponding author.
